# Plant Nitric Oxide Signaling under Drought Stress

**DOI:** 10.3390/plants10020360

**Published:** 2021-02-13

**Authors:** Su-Ee Lau, Mohd Fadhli Hamdan, Teen-Lee Pua, Noor Baity Saidi, Boon Chin Tan

**Affiliations:** 1 Centre for Research in Biotechnology for Agriculture, University of Malaya, Kuala Lumpur 50603, Malaysia; suee@um.edu.my (S.-E.L.); teenleepua@um.edu.my (T.-L.P.); 2 Department of Cell and Molecular Biology, Faculty of Biotechnology and Biomolecular Sciences, Universiti Putra Malaysia, Serdang 43400, Malaysia; norbaity@upm.edu.my; 3 School of Biological Sciences, The University of Hong Kong, Pokfulam Road, Hong Kong, China; fadhli@hku.hk

**Keywords:** abiotic stress, crop improvement, drought, nitric oxide, S-nitrosylation, signaling molecule, water deficit

## Abstract

Water deficit caused by drought is a significant threat to crop growth and production. Nitric oxide (NO), a water- and lipid-soluble free radical, plays an important role in cytoprotection. Apart from a few studies supporting the role of NO in drought responses, little is known about this pivotal molecular amendment in the regulation of abiotic stress signaling. In this review, we highlight the knowledge gaps in NO roles under drought stress and the technical challenges underlying NO detection and measurements, and we provide recommendations regarding potential avenues for future investigation. The modulation of NO production to alleviate abiotic stress disturbances in higher plants highlights the potential of genetic manipulation to influence NO metabolism as a tool with which plant fitness can be improved under adverse growth conditions.

## 1. Introduction

The loss of millions of hectares of arable lands to various stresses every year continues to impose a major threat to agriculture. Almost 90% of all arable lands have been exposed to major environmental stresses, such as salinity, drought, extreme temperature, heavy metals, and ultraviolet radiation [1]. Of these, water deficit caused by drought is amongst the main threats to crop growth and production worldwide. In the US alone, the combined effect of drought and increased temperature has caused a total loss of about USD555 billion for the past 10 years (2010–2019) in agricultural production [2]. The effect of drought is even more devastating in developing countries whose economies largely rely on their agricultural output. According to the Food and Agriculture Organization of the United Nations (FAO), 30% of agricultural losses (USD ~29 billion) due to drought was recorded in developing countries in the period of 2005–2015 [3].

Drought conditions cause a disruption to the cellular redox homeostasis in plants, leading to oxidative stress and cell injury [4]. Plants respond and adapt to drought stress by changing their morphology, biochemistry, physiology, and molecular mechanisms [5]. One of the major signaling molecules present in the cascade of this stress adaptation response is nitric oxide (NO), a water- and lipid-soluble free radical and a gaseous, redox-related signaling molecule that is rapidly produced by multiple hormonal and environmental stimuli [6]. NO regulates and maintains the level of reactive oxygen species (ROS) by inducing transcriptional changes of various targets involved in plant defense and cell death, translocation, signal transduction, and ROS metabolism [7].

Some studies have supported the roles of NO in drought adaptation, but little is known about this important molecular amendment in modulating signaling in abiotic stresses. Studies regarding the origin and production of NO during water deficit, its sensing, and transduction as well as its physiological and molecular processes for the amelioration of drought stress remain scarce. Continued efforts in examining the roles of NO during plant adaptation to drought stress and its detection and measurement methods are, therefore, crucial for facilitating breeding and crop improvement programs to counter the challenges faced by the agricultural industry. In this review, we provide an overview of NO biosynthesis and its roles and regulatory mechanisms in response to drought stress. We also highlight the technical challenges of NO detection and measurements and suggest potential avenues for future investigation.

## 2. Nitric Oxide: Background

### 2.1. Source and Biosynthesis of NO in Plants

**Figure 1:**
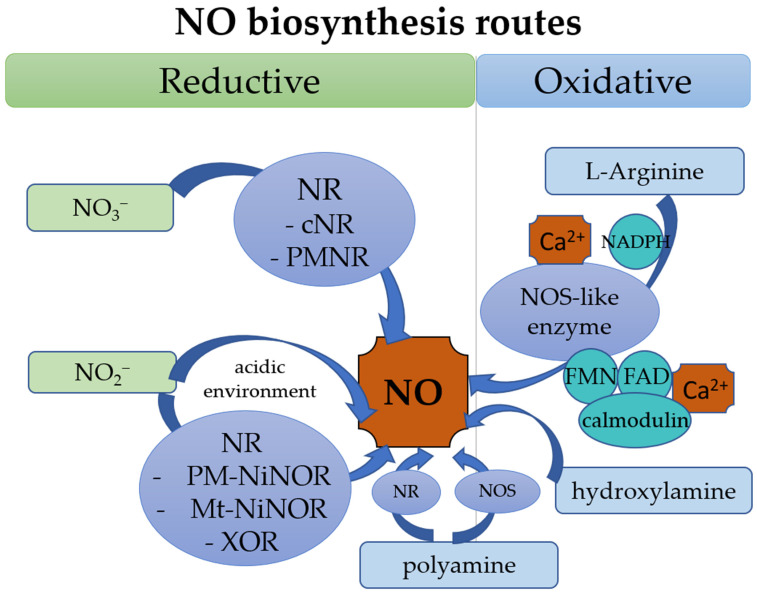
Schematic diagram showing the major routes of nitric oxide (NO) biosynthesis. NO synthesis occurs through (I) reductive mechanism or (II) oxidative mechanism. (I) NO reductive mechanism mostly occurs in plasma membrane, chloroplasts, apoplast, peroxisomes, cytoplasm, and mitochondria through reduction of nitrate (NO_3_^−^) or nitrite (NO_2_^−^) by nitrate reductase (NR). NO production through nitrate substrate is reduced by cytosolic nitrate reductase (cNR) and plasma membrane-bound nitrate reductase (PMNR), whereas NO production through nitrite substrate is reduced by plasma membrane-bound nitrite: NO reductase (PM-NiNOR), mitochondrial electron transfer chain-dependent enzymatic nitrite: NO reductase (Mt-NiNOR), XOR. In an acidic environment, nitrite produces NO through several reversible reactions. Polyamines induce NO production through reductive and oxidative mechanisms. (II) The oxidation of L-Arg takes place in chloroplasts and leaf peroxisomes. In chloroplasts, the oxidation of L-Arg requires nicotinamide adenine dinucleotide phosphate (NADPH) and calcium ions (Ca^2+^), whereas in leaf peroxisomes, the oxidation of L-Arg requires flavin mononucleotide (FMN), flavin adenine dinucleotide (FAD), calmodulin, and Ca^2+^. Hydroxylamine acts as a substrate in the oxidative mechanism; however, its pathway in NO production remains unclear. NOS, NO synthase.

NO biosynthesis has been well characterized in mammals. It is mainly synthesized through NO synthase (NOS) activity [8]. In plants, NO can be biosynthesized via reductive and oxidative routes [9]. The most thoroughly researched NO biosynthetic pathway in plants is the reduction of nitrite, either enzymatically or nonenzymatically. Enzymes that have been demonstrated to catalyze the generation of NO from nitrites are nitrate reductase (NR) [10], the membrane-bound nitrite NO reductase (NiNOR) [11], and peroxisomal xanthine oxidoreductase (XOR) [12]; mitochondrial electron transfer chain-dependent enzymatic nitrite reduction has also been reported to be capable of this [13]. In contrast, nonenzymatic reduction has been reported to occur in the apoplast of barley aleurone layers under acidic environments with the presence of high concentrations of nitrate (NO_2_^−^) [14]. The oxidative route of NO production relies on the oxidation of aminated molecules, such as L-arginine(L-Arg) by the L-Arg-dependent NOS-like enzyme [15], polyamines [16], and hydroxylamine [17]. The routes of NO biosynthesis are illustrated in Figure 1.

### 2.2. NO Donors

**Figure 2:**
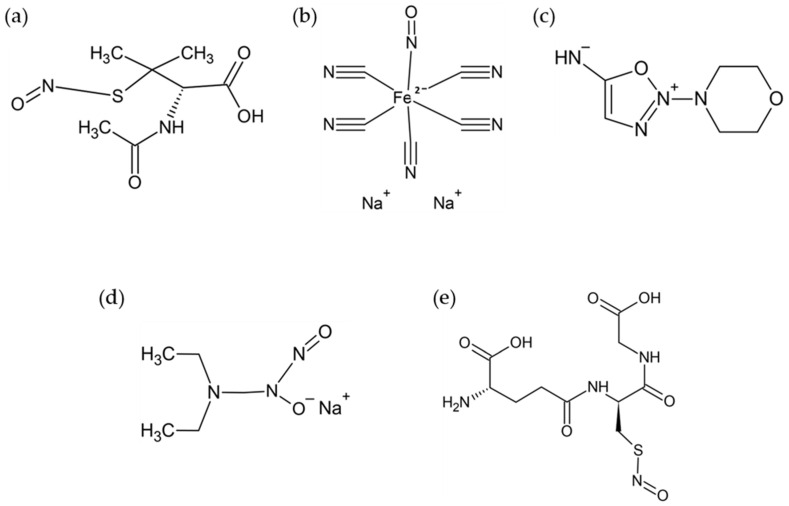
Chemical structures of nitric oxide (NO) donors. (**a**) S-nitro-N-acetylpenicillamine (SNAP), (**b**) sodium nitroprusside (SNP), (**c**) 3-morpholinosydnonimine (SIN-1), (**d**) NONOates, and (**e**) S-nitroglutathione (GSNO).

Synthetic NO donors are active substances that release NO. They have been widely used as experimental tools to explore the roles of endogenous NO in plant adaptation to abiotic and biotic stresses. Examples of NO donors are sodium nitroprusside (SNP), S-nitro-N-acetylpenicillamine (SNAP), 3-morpholinosydnonimine (SIN-1), S-nitroglutathione (GSNO), NONOates, and organic NO_3_^−^ (Figure 2) [18]. Among the three most used NO donors, namely SNAP, GSNO, and SNP, the NO generation rate is the highest in SNAP, followed by GSNO and SNP. In contrast, SNP has the longest half-life in the solution (12 h) compared to GSNO (7 h) and SNAP (3 h). In terms of photosensitivity, SNP is much higher when compared with GSNO and SNAP [19]. Among these NO donors, SNP is widely used in plant research because of its stability and its stability to act as a long-lasting NO reservoir [13].

NO donors are either supplemented to the roots in growth media or through fertigation [20], which is sprayed onto the plants’ leaves [21] with the aid of nanoparticles [22,23], or through a seed-priming agent [24,25]. The mechanisms of action of NO are yet to be fully revealed as it is highly dependent on various factors, such as plant species, age, size, source, concentration of NO donor, treatment duration, and methods of application [26]. For instance, application of 0.2 mmol/L SNP alleviated drought tolerance in wheat seedlings, whereas a concentration of SNP that was 10 times higher led to excessive generation of ROS and low antioxidant activities [27]. This observation was in agreement with the study by Cao et al. [11], where root oxidative damage caused by water stress was successfully alleviated by a lower concentration of SNP (less than 20 μmol/L), whereas a higher concentration of SNP (more than 40 μmol/L SNP) affected the root growth, suggesting that high NO contents might be toxic to plants and cause cellular damage.

### 2.3. Functions of NO in Plants

NO was initially considered as an air pollutant. This molecule has also been reported to pose an inhibitory effect on plant growth by fragmenting DNA and damaging membranes [28], reducing the photosynthesis level in oat and alfalfa [29], and lowering respiration in carrot cell suspensions [30]. From simply an air pollutant, NO has now become a vital redox signaling molecule.

NO and its derived molecules (reactive nitrogen species, RNS) are ubiquitous in plants. Other RNSs include peroxynitrite (ONOO−), S-nitrosoglutathione (GSNO), and S-nitrosothiols (SNOs). NO and RNS are involved in various developmental and physiological processes, such as seed germination and dormancy [31], shoot and root growth [32], flowering [33], fruit ripening [34], yield [35], stomatal closure [36], formation of guard cells [37], senescence [38], photosynthesis regulation [39], mitochondria functionality [30], gravitropism [40] and pollen tube growth [41]. The way by which NO and RNS exert their function is still largely unknown. It was shown that NO exerts part of its effects through post-translational modifications (PTMs), such as tyrosine nitration, S-nitrosylation, and metal nitrosylation. The details of plant proteins modified by NO at the post-translational level will be discussed in Section 4.

It is now recognized that NO protects against damage provoked by oxidative stress due to biotic [42,43] and abiotic stresses [44]. ROS can be produced under both normal and stressed conditions. However, plants produce more ROS accumulation under stress conditions [45]. NO may act as a detoxifier of ROS by minimizing its harmful effect towards plants [46]. This phenomenon was observed in plants challenged with metal toxicity [47], high temperature [48], salinity [49], and chilling stress [50]. The roles of NO in alleviating drought stress will be described in Section 3.

## 3. NO and Drought Stress

### 3.1. Impacts of Drought on Plants

Drought stress adversely impacts plant growth and development at physiological, biochemical, and molecular levels. It disturbs the appearance of plants by causing chlorosis and necrosis signs [21]; prolongs the duration of primordia initiation; and reduces the number of shoots per explant, shoot length, leaf number, leaf area [51], fruit yield, and plant biomass [26]. Besides the aboveground plant parts, underground parts like roots also respond to drought by changing their root length to become either shorter [52] or longer for efficient uptake of soil moisture [51].

At the physiological level, drought stress reduces stomatal conductance and increases transpiration rate [23]. This causes an unbalanced transpiration rate and results in low leaf relative water content (LRWC) [53]. Water potential, water use efficiency, and hydraulic conductivity in drought-stressed plants are remarkably reduced, whereas leaf venation density is increased, indicating that water deficit negatively interferes with the water transportation in leaves [37]. Photosynthesis, an important biological process for plant survival, is significantly influenced by drought. These include the reduction of leaf carbon dioxide assimilation, ribulose-1,5-bisphosphate (RuBP) regeneration [22], chlorophyll and carotenoid contents, and accessory pigments [53].

At the biochemical level, drought stress induces a high level of ROS, which can lead to cellular damage. To respond, plants close their stomata to limit gas exchange. The reduced carbon dioxide levels in chloroplasts decrease NADP+ regeneration, causing higher leakage of electrons to oxygen (O_2_) to form superoxide anions (O_2_^•−^) and subsequently, leading to the accumulation of ROS. If drought becomes severe, the reduced rate of chloroplast ATP synthesis increases the mitochondrial respiration rate, which eventually induces the accumulation of more ROS in the mitochondria [54]. In plants, increased lipid peroxidation (MDA); lipoxygenase (LOX) activity; aldehyde, proline, cysteine, and electrolyte leakage; and hydrogen peroxide (H_2_O_2_) production have been used to assess the increase of ROS production under drought [55]. Several Calvin–Benson cycle enzyme activities, such as RuBisCo, GAPDH, and phosphoribulokinase, have also been shown to be affected by drought stress [56].

At the molecular level, several genes and signal transduction pathways have been found to be important in drought stress responses. These include abscisic acid (ABA), strigolactone, lipid-derived signal, ROS, NO, transcription factors, and soluble sugars [57]. Plants perceive the external drought stimuli via sensors on the membrane and transmit them down the signals through multiple signal transduction pathways. This causes the changes in drought-responsive gene expression and adaptation. Drought-responsive genes are regulated by complex mechanisms, including the transcriptional cascades. Among the transcription factors, AP2/EREBP, WRKY, MYB, NAC, DREB, HSFF, and bZIP are commonly reported transcription factor families in drought stress response [58]. There are two groups of drought-responsive genes, i.e., ABA-dependent and ABA-independent. Many drought-responsive genes respond to ABA. These ABA-dependent genes encode the late embryogenesis abundant (LEA) proteins (the most reported ABA-dependent), enzymes (involved in the generation of osmolytes, detoxification, and metabolisms), transporters (ion transporters and channel proteins), transcription factors, and protein kinases. Some genes, however, do not respond to ABA. DREB2 proteins, members of the AP2/ERF transcription factor family, are among the important proteins in the ABA-independent pathway in response to drought stress conditions [59]. Most photosynthesis genes, such as *Psas*, *Psbs*, and *Pets*, have also been reported to be downregulated in drought-stressed plants, whereas antioxidant enzyme-related genes, including *GSTs*, *PODs*, *CATs*, *SODs*, and *GPXs*, as well as genes encoding the respiratory burst oxidase protein (RBOH), have been found to be upregulated [46]. Other drought-responsive genes involved in transportation (*aquaporin* (*PIP*) gene), lipid and sugar metabolisms, wax synthesis, cell wall regulation, and osmotic adjustment have also been reported [60]. Besides changing the gene expression, drought stress also induces genotoxic alterations, such as DNA damage and mutational events [56].

To combat these adverse effects of drought stress, plants have some inbuilt mechanisms, such as enzymatic (superoxide dismutase (SOD), peroxidase (POD), catalase (CAT), glutathione peroxidase (GPX), and ascorbate peroxidase (APX)) and nonenzymatic antioxidant enzymes (phenolic compounds, flavonol, anthocyanin, tocopherol, ascorbic acid, carotenoids, and those involved in secondary metabolic biosynthesis like phenylalanine ammonia-lyase (PAL) and tyrosine ammonia-lyase (TAL)).

### 3.2. The Involvement of NO in Drought Tolerance

The role of NO in mitigating drought stress effects has been observed in several plant species, including grains, legumes, fruit trees, medicinal plants, and vegetables (Table 1). The production of NO depends on the drought stress level, exposure duration, and plant growth stages. For instance, the accumulation of NO in *Cucumis sativus* increased from 75-fold within the first 10 h of drought exposure to 190-fold after 17 h of drought exposure compared to well-watered plants [61]. Different types of nitrogen nutrition supplied to plants have been reported to influence the rapidness of endogenous NO production. Cao et al. [11] reported that rice supplemented with ammonium (NH_4_^+^) induced endogenous NO production after 3 h of drought treatment, whereas those supplemented with NO_3_^−^ induced NO production after 6 h of drought treatment. Their findings indicate that NH_4_^+^ is more effective than NO_3_^−^ in alleviating water stress, probably due to the early NO burst which might be triggered by NOS-like enzymes in roots.

Exogenous application of NO-induced stomatal closure in *Tradescantia* sp., *Salpichroa organifolia*, and *Vicia faba* [62]. The saturated net carbon dioxide assimilation rate, stomatal conductance, substomatal carbon dioxide concentration, and transpiration rate were also increased after NO treatment [37]. By applying the NO scavenger 2-phenyl-4,4,5,5,-tetramethylimidazoline-1-oxyl 3-oxide (PTIO), stomatal closure was inhibited, supporting the notion that NO is induced endogenously and most likely could act as a signaling element to facilitate stomatal closure. In addition, NO may promote the formation of guard cells and influence the distribution of stomata in the abaxial leaf [63].

**Table 1:** Recent studies of nitric oxide on drought stress.

Plant	Drought Imposition and Duration	Source of NO	Concentration (µmol/L)	Application Method	Response under Water Deficit Condition	Reference
Alfalfa (*Medicago sativa* L.)	10% of PEG for 7 d	SNP	100	Seeds were germinated on filter papers containing treatment solutions (SNP and PEG)	Increment of the fresh weight, LRWC, chlorophyll content, proline content, soluble sugar contents and antioxidant enzyme activities (NR, SOD, POD, CAT, and APX), and reduction of root length, MDA level, differentially expressed genes involved in antioxidative defense system, photosynthesis, hormonal signal transduction, carbohydrate metabolism, and secondary metabolism	[46]
	Withholding water for 6 d	NOSH or NOSH-A	100	Foliar spray	Improved acclimation to drought stress and improved the recovery after re-watering by reducing lipid peroxidation and proline accumulation levels	[21]
Apple rootstocks (*Malus* spp.)	Withholding water for 7 d	SNP	50, 100, 200, 300, and 400	Foliar spray (5 times per d)	Protection of *Malus* seedlings from drought-induced oxidative damage by enhancing antioxidant enzyme activities and photosynthetic performance	[64]
Banana (*Musa acuminata* cv. Berangan)	5% PEG for 9 d	SNP	5	SNP was supplied in liquid MS medium	Increment of the dry weight, number of roots formed, and antioxidant enzyme activities (SOD, CAT, APX, and GR); reduction of the percentage of yellow leaves	[65]
Broccoli (*Brassica oleracea* L.)	60% field capacity for 21 d	SNP	20	Pre-sowing (seeds were soaked with SNP for 15 h) or foliar spray once	Enhancement of the fresh and dry biomass of shoot, shoot length, chlorophyll contents, GB, total phenolics, total soluble proteins, and activities of SOD and POD enzymes under water deficiency	[31]
Canola (*Brassica napus* L. Dunkeld and L. Cyclone)	60% field capacity for 21 d	SNP	20	Foliar spray once	Upregulation of the oxidative defense system, osmoprotectant accumulation, and minimizing the lipid peroxidation.	[66]
Common bean (*Phaseolus vulgaris* L. cv. IAC Mileˆ- nio)	PEG (osmotic potential of - 0.3 MPa) for 12 and 17 d	GSNO	50, 100, 500, 1000, and 2000	Pre-sowing (seeds were soaked with GSNO for 1 min)	Improvement of seed germination and increment of initial root growth	[25]
Crambe (*Crambe abyssinica*)	50% of the maximum water holding capacity for 32 and 136 h	SNP	75, and 150	Foliar spray (4 consecutive days at 24-h intervals)	Increment of water potential, osmotic potential, NR activity, photosynthetic rate, stomatal conductance, transpiration rate, photochemical efficiency of PSII (Fv/Fm), the effective quantum yield of photosystem II (ΦPSII), electron transport rate (ETR), initial fluorescence (F0), quantum yield of regulated energy dissipation (ΦNPQ), chlorophyll a and b content, antioxidant enzyme activities (SOD, CAT, APX, and GR) and reduction of MDA content, H_2_O_2_ level and O_2_ concentration in leaves	[67]
Cucumber (*Cucumis sativus* L.)	0.05% of PEG for 6 d	SNP	1, 10, 50, and 100	Explants were placed on filter papers moistened with SNP	Increment of root number and length	[68]
European searocket (*Cakile maritima* Scop.)	Withholding water for 2, 7, and 14 d	SNP	100	Seedlings were pre-treated with Hoagland medium containing SNP for about 20 d	Improvement of growth activity, increment of chlorophyll and carotenoids contents, LRWC, proline content, P5CS protein accumulation, SOD and CAT enzyme activity, and reduction of osmotic potential, MDA content, and EL	[69]
Indian mustard (*Brassica juncea* cv. Varuna)	10% of PEG for 4 d	SNP	100	Seedlings were treated with Hoagland medium containing SNP for 4 d	Increment of LRWC, chlorophyll content, net photosynthestic rate, internal CO_2_ concentration, stomatal conductance, transpiration rate, PSII efficiency, photochemical quenching, non-photochemical quenching, electron transport chain, RuBisCo, GAPDH, phosphoribulokinase, ATP-S, SAT activities, and genomic DNA template stability and reduction of thiobarbituric acid reactive substances, EL and OH^−^ content	[56]
Indian mustard (*Brassica juncea* cv. Pusa Jagannath and cv. Varuna)	10% of PEG for 4 d	SNP	100	Seedlings were treated with Hoagland medium containing SNP for 4 d	*Brassica juncea* cv. Pusa Jagannath had the antioxidant protection mainly through the accumulation of nonenzymatic antioxidants, whereas *Brassica juncea* cv. Varuna showed tolerance by the enhancement of both enzymatic and nonenzymatic antioxidant activities	[53]
Marjoram (*Origanum majorana* L. German type)	70% depletion of available soil water for 95 d	SNP	30 and 60	Foliar spraying using a hand-atomizer on days 35, 50, 65, and 80	Improved water use efficiency, increased plant biomass and essential oil yield and percentage, phytopharmaceuticals of herbal extract (ascorbic acid, total soluble phenol, anthocyanin, and flavonoid), antioxidant enzyme activities (CAT and POD), plant extract reducing power and reduction of H_2_O_2_, MDA, protein carbonyl group and percentage of EL	[70]
Milk thistle (*Silybum marianum*)	Withholding water for 14 d	SNP	100 and 200	Whole plants were sprayed with SNP 4 times using a wheeled plot sprayer	Increment of photosynthetic rate, chlorophyll a, b and carotenoid content, and seed yield	[71]
Perennial ryegrass (*Lolium perenne* L.)	Withholding water for 23 d	GSNO	100	Foliar spray every 2 d	Increment of total chlorophyll and carotenoids content, 1-SST activity associated with increased fructan content, GR activity, and S-nitrosothiols content and reduction of MDA content, H_2_O_2_ level, and ∙OH levels	[72]
Persian shallot (*Allium hirtifolium*)	2, 4, 8 and 16 mmol/L of PEG for 16 weeks	SNP	10, 40, and 70	SNP was supplied in MS medium	Increment of regeneration rate, LRWC content, photosynthetic pigments, antioxidant enzyme activity (SOD and APX), proline and allicin accumulation, and reduction of H_2_O_2_, and MDA content in leaves	[73]
*Physalis angulata*	80% and 20% field capacity for 20 d	SNP	25, 50, 75, and 100	Foliar spray with SNP twice per experiment	Low concentrations of SNP have shown to mitigate negative effects of water deficit, improving photosynthetic rates, maintenance of leaf water potential, and growth	[26]
Rapeseed (*Brassica napus* cv. BINA Sarisha 3)	10% and 20% of PEG for 2 d	SNP	500	Seedlings were pre-treated with Hyponex solution containing SNP	Improvement of the levels of nonenzymatic antioxidant pool, glyoxalase system, and upregulation of antioxidant enzyme activities	[74]
Safflower (*Carthamus tinctorius* L.)	20% of field capacity for 21 d	SNP	25	Whole plants were sprayed with SNP (1 d before and 7 d after drought stress treatments)	Improvement of the growth of aerial part, increment of chlorophyll content, and reduction of anthocyanin, flavonoid and phenol contents, and root length	[51]
Rice (*Oryza sativa* L. ‘Zhongzheyou No. 1’ hybrid *indica*)	10% of PEG for 21 d	SNP	20	Seedlings were treated with SNP solution once	Increment of antioxidant activities (CAT, APX, and SOD), and reduction of O_2_^•−^, H_2_O_2_, OH^−^, and ONOO^−^ content, MDA, and carbonyl levels in root	[11]
Soybean (*Glycine max*)	20% water in the soil for 30 d	SNP	100	Foliar spray in 3 d intervals	Increment of photosynthesis and biomass accumulation	[37]
	5, 10, and 15% of PEG for 21 d	SNP	100	Whole plants were sprayed with SNP using an atomizer on alternate day	Increment of FW, SOD, CAT, POX, APX, PPO enzyme activities, phenylalanine ammonia-lyase, and tyrosine ammonia-lyase activities, total phenol, flavonol, and tocopherol content, and reduction of MDA content, aldehyde, H_2_O_2_ content, LOX activity, and EL	[55]
	15% of PEG for 2 d	SNP	50, 100, 200, 400, and 600	SNP was supplied in Hoagland medium	Positively regulated the transcription of genes encoding Cyt-G6PD (*GPD5*, *G6PD6,* and *G6PD7*)	[75]
*Stevia rebaudiana* Bertoni	5%, 10% and 15% of PEG for 20, 40 and 60 d	SNP	50, 100, 250, and 500	SNP was supplied in MS medium	Increment of shoot length, shoot number, and leaf number	[52]
Sugarcane (*Saccharum* spp.)	PEG treatment for 7 d (−0.75 MPa)	NO_3_^−^:NH_4_^+^ ratios 100:0 and 70:30	5	NO_3_^−^:NH_4_ was supplied in nutrient solution; plantlets were cultured in the medium for 2 weeks before drought stress treatment	Increment of photosynthetic rate, stomatal conductance, root growth, and reduction of ROS accumulation in plants treated with more nitrate	[32]
	PEG treatment for 5 d (-0.75 MPa)	GSNO	10, 100, 500, and 1000	Foliar spray twice per day	Increment of photochemical activity, LRWC, leaf, and root dry matter	[76]
Thyme (*Thymus serpyllum* Serpolet and *T. Vulgaris* L.).	80, 60 and 40% field capacity	SNP	50, 100, 150, and 200	Whole plants were sprayed with SNP 3 times per experiment (prior to flowering stage, at 50% flowering, and at full bloom)	Increment of proline accumulation and reduction of some antioxidant activities which increased drought tolerance	[77]
Tomato (*Lycopersicon esculentum* Mill.)	Withholding water for 7 d	SNP	50 and 100	Foliar spray on alternate day after 1 d of water holding	Improvement of drought tolerance by increasing SOD activity, reducing H_2_O_2_, and other physiological processes (increased leaf number, average number of flower clusters per plant, LRWC, and lycopene content)	[78]
Watermelon (*Citrullus lanatus* var. lanatus KAR 98)	15% of PEG for 10 d	SNP	100	SNP was supplied in Hoagland medium; the medium was replaced every 3 d	Increment of root length, APX, GR antioxidant activities, and reduction of MDA content, OH^−^, osmotic potential, and EL	[79]
Wheat (*Triticum aestivum* L. cv. Pishgam)	Soil moisture at 75% and 50% for 6 weeks	SNP	100	Foliar spray	Increment of seedling length, SOD enzyme activity, total soluble proteins, net photosynthetic rate, and intercellular carbon dioxide concentration and reduction of H_2_O_2_, and MDA contents	[23]
Wheat (*Triticum aestivum* L. cv. Prodip)	15% and 30% of PEG for 9 d	SNP	0.5	SNP was supplied in Hyponex solution	Enhancement of the antioxidant defense system (both nonenzymatic and enzymatic components) in drought-stressed seedlings, endogenous NO content, glyoxalase system, and reduction of methylglyoxal content, which restored the LRWC, and further increased the proline content	[80]
Wheat (*Triticum aestivum* cv. Jing 852)	7.5% of PEG for 5 d	SNP	5	SNP was supplied in Hoagland medium	Increment of lateral and primary root length	[81]
Wheat (*Triticum aestivum* cv. BARS-2009 and *Triticum aestivum* cv. Mairaj-2008)	35% of water holding capacity till physiological maturity	SNP	50, 100, and 150	Foliar spray (7 d before drought stress treatment) using a hand sprayer	Increment of yield, chlorophyll contents, accumulation of soluble phenolics, proline, and GB, and reduction of MDA contents	[82]
Wheat (*Triticum aestivum* cv. 98SN146 and *Triticum aestivum* cv. Longchun22)	PEG for 24 h	SNP	100	Seedlings were pre-sprayed with SNP (12 h before drought stress treatment)	Increment of cyanide-resistant respiration, pyruvate content, *Alternative oxidase* gene (*AOX1a*), and alternative pathway	[83]
White clover (*Trifolium repens*)	PEG (-0.3 Mpa) for 8 d	SNP	50	SNP was supplied in Hoagland medium; plantlets were cultured in the medium for 3 d before drought stress treatment	Effectively mitigated water stress damage; changes of metabolic profiles, and associated metabolic pathways which could contribute to enhance stress tolerance	[84]

APX, ascorbate peroxidase; CAT, catalase; EL, electrolyte leakage; ETR, electron transport rate; F0, initial fluorescence; GAPDH, glyceraldehyde-3-phosphate dehydrogenase; GB, glycine betaine; GSNO, S-nitrosoglutathione; H_2_O_2_, hydrogen peroxide; LRWC, leaf relative water content; MDA, malondialdehyde; MS, Murashige and Skoog medium; NOSH-A, NOSH-aspirin; NR, nitrate reductase; O_2_^•−^, superoxide anion radical; O_2_, oxygen; OH^−^, hydroxyl radical; ONOO-, peroxynitrite; PEG, polyethylene glycol; PPO, polyphenol oxidase; POD, peroxidase; PRK, phosphoribulokinase; RuBisCo, ribulose 1,5-bisphosphate carboxylase/oxygenase; SNP, sodium nitroprusside; SOD, superoxide dismutase; ΦPSII, photosystem II; ΦNPQ, quantum yield of regulated energy dissipation.

At the molecular level, NO induces the expression of stress-related genes in response to drought stress. For instance, NO positively regulated the transcription of stress-adaptation-related genes, i.e., *Cyt-G6PD* (*GPD5*, *G6PD6*, and *G6PD7*) in soybean roots [75]. Zhao et al. [46] found that more than 2000 genes were differentially expressed in drought-stressed alfalfa plants treated with or without NO. These differentially expressed genes are involved in several critical cellular and physiological processes, such as the antioxidative defense system, photosynthesis, hormonal signal transduction, carbohydrate metabolism, and secondary metabolism [46]. When Shi et al. [85] exposed the rat neuronal NO synthase (nNOS)-expressing transgenic *Arabidopsis* to drought, they found that the gene expression profile (184 genes upregulated and 263 downregulated) and antioxidant enzymatic activities were changed. Endogenous NO has also been found to improve the stability of genomic DNA in drought-stressed plants, such as Indian mustard [56], *Brassica rapa* [86], and *Oryza sativa* [87]. These findings highlight the importance of NO in drought stress tolerance. However, the details regarding NO signaling and its crosstalk with other molecules are still unclear and require further exploration.

### 3.3. The Involvement of NO in the Antioxidant System

**Figure 3:**
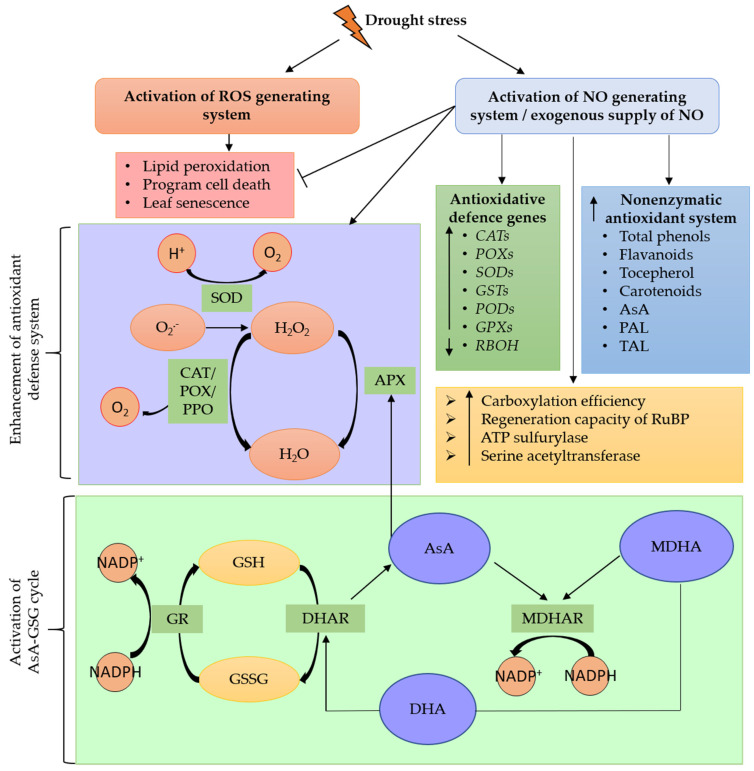
Activation of enzymatic and nonenzymatic antioxidant systems by nitric oxide (NO) to detoxify cellular reactive oxygen species (ROS) induced by drought stress. APX, ascorbate peroxidase; AsA, ascorbate; ATP, Adenosine triphosphate; CAT, catalase; DHA, dehydroascorbate; DHAR, dehydroascorbate reductase; GPX, glutathione peroxidase; GR, glutathione reductase; GSH, reduced glutathione; GSSG, oxidized glutathione; GST, glutathione S-transferase; H^+^, hydrogen ion; H_2_O_2_, hydrogen peroxide; MDHA, monodehydroascorbate; MDHAR, monodehydroascorbate reductase; NADP^+^, nicotinamide adenine dinucleotide phosphate; NADPH, reduced form of nicotinamide adenine dinucleotide phosphate; O_2_^•−^, superoxide anion; PAL, phenylalanine ammonia-lyase; POD and POX, peroxidases; PPO, polyphenol oxidase; RBOH, respiratory burst oxidase protein; RuBP, ribulose-1,5-bisphosphate; SOD, superoxide dismutase; TAL, tyrosine ammonia-lyase.

NO regulates the levels of cellular ROS and toxicity through two main pathways, enzymatic and nonenzymatic antioxidant enzymes (Figure 3) [88]. Majeed et al. [35] reported that the level of MDA and H_2_O_2_ in drought-stressed maize was reduced by almost 50% after NO treatment [35]. The reduction of H_2_O_2_ and O_2_^ˉ^ might be due to the enhanced antioxidative defense activities of NO [84]. Activities of SOD, CAT, and APX have been observed to be influenced by NO. With NO treatment, Rezayian et al. [55] found that activities of SOD, CAT, APX, PPO, and POX in soybean under a mild drought condition (5% polyethylene glycol (PEG)) were higher than under moderate (10% PEG) and severe (15%) drought conditions. The application of an NO donor, i.e., SNP, increased CAT activity in PEG-induced drought-stressed rice, whereas PEG alone decreased CAT activity [11]. At the mRNA level, it has been shown that endogenously induced or exogenously introduced NO triggered upregulation of antioxidative defense genes, including *OsCATA*, *OsCATB,* and *OsPOX1* in rice [89]; *Cu/Zn-SOD* in maize [90]; *GSTs, PODs*, *CATs*, *SODs*, and *GPXs* in alfalfa, but downregulation of *RBOH* gene [46].

It is well known that drought stress could activate leaf senescence and programmed cell death. NO has been reported to act as a negative regulator of leaf senescence and programmed cell death. During leaf senescence, a gradual decline in antioxidants and an increase in ROS are detected. NO could delay leaf senescence by enhancing CAT and SOD activities and inducing the production of other enzymes, such as s-transferase, alternative oxidase, and glutathione (GSH) [91]. To mitigate oxidative damage caused by ROS, NO increases the carboxylation efficiency, regeneration capacity of RuBP, as well as ATP-sulfurylase and serine acetyltransferase activities [56]. As a signal mediator, NO is also involved in cold- and starvation-stress-induced programmed cell death in barley [92]. Jiang et al. [93] showed that the level of NO began to accumulate and achieved the highest value at the loading stage of cryopreservation in *Dendrobium*.

Nonenzymatic antioxidants also play a vital role in scavenging ROS. Among the non-enzymatic antioxidants, AsA, and GSH have the strongest protective effect against drought-induced oxidative stress [45,94]. It has been reported that NO increased the accumulation of phenolic compounds, including total phenol, flavonol, and tocopherol, in soybean when exposed to water deficit conditions [55]. Both important phenolic biosynthetic enzymes, PAL and TAL, were also highly expressed by NO treatment [55]. Similar findings were reported in drought-stressed broccoli [31] and wheat [82]. Taken together, NO could act as a signaling molecule to maintain ROS homeostasis and enhance drought stress tolerance in plants by regulating antioxidant machinery.

### 3.4. Synergistic Relationship of NO with Other Signaling Molecules

NO mediates its action in a concerted way with other signaling molecules, including hydrogen sulfide (H_2_S), H_2_O_2_, calcium (Ca^2+^), melatonin, and polyamines (PAs). H_2_S is an important signaling molecule with effects on many biological processes, such as growth and development, as well as defense responses in plants [95]. Emerging evidence indicated that H_2_S interacts with other signaling molecules to modify their signal. It is believed that the mechanisms of action of H_2_S are via a PTM of proteins, called persulfidation [96]. In this modification, reactive cysteine residues on target proteins are modified by converting the thiol group (-SH) into a persulfide group (-SSH). These persulfidated proteins are believed to be involved in cellular functions and metabolic pathways, as well as protection against overoxidation.

The function of H_2_S in stomatal closure under drought stress has been shown to be mediated by NO. By exogenously applying H_2_S to *Vicia faba*, Garcia-Mata and Lamattina [62] found that H_2_S induced stomatal closure through the regulation of ATP-binding cassette (ABC) transporters. This effect can be reverted by cPTIO (2-(4-carboxyphenyl)-4,4,5,5 tetramethylimidazoline-1-oxyl-3-oxide) (an NO-specific scavenger), suggesting the synergistic effect of NO and H_2_S. Antoniou et al. [21] found that alfalfa plants adapted to drought stress conditions after being pretreated with NO and H_2_S. They indicated that NO and H_2_S treatment improved vitality, turgor, greening of leaves, stomatal conductance, and chlorophyll content [21]. Besides improving the morphophysiology of plants, NO, together with H_2_S, has also been found to improve antioxidant enzyme activities, such as those of APX, GR, POX, SOD, and CAT, and enhance the accumulation of osmolytes (proline and glycine betaine). These effects were reversed by the NO scavenger, cPTIO, indicating the protective role of both molecules against drought stress [97].

H_2_O_2_ is a signaling molecule that regulates plant metabolism by cooperating with other signaling molecules. Its production can be stimulated by NO [98]. Together, they modulate the accumulation of osmolytes, regulate ABA-induced antioxidant enzyme activities, induce stomatal closure, protect mesophyll cell ultrastructure, and improve photosynthesis [99,100,101,102]. When priming the wheat seedlings with NO and H_2_O_2_, Wang et al. [99] found that the tolerance level of pretreated wheat seedlings was enhanced. The accumulation of proline and glycine betaine was also increased [99].

Ca^2+^ is a secondary messenger regulating many physiological processes in plants. NO has been shown to govern Ca^2+^ homeostasis, in addition to almost all types of Ca^2+^ channels and transporters. In plants, NO increases the cytosolic Ca^2+^ concentration when under stress conditions. The increased cytosolic Ca^2+^ concentration, on the other hand, induces NO biosynthesis through calcium-binding proteins, suggesting that NO and Ca^2+^ can synergistically improve plant tolerance to adverse environmental conditions like drought. Several studies have been carried out to elucidate the relationship between these two signaling molecules. NO was first found as a Ca^2+^-mobilizing messenger in plants when studies evaluated the ability of NO donors in increasing the intracellular Ca^2+^ concentration [103,104]. Lamotte et al. [103] showed that diethylamine NONOate (NO donor) induced a transient rise in cytosolic Ca^2+^ concentration in transgenic *Nicotiana plumbaginifolia*, whereas application of the NO scavenger cPTIO and NOS inhibitors decreased the cytosolic Ca^2+^ concentration. However, Rodríguez-Serrano et al. [105] reported that the production of NO was increased by addition of Ca^2+^. Two mechanisms of action of NO on Ca^2+^ channel and transporter activities have been reported: a cGMP-dependent mechanism and a cGMP-independent mechanism. The mechanism of the cGMP-dependent pathway is complex. At least three processes have been reported to date: (i) cGMP directly activates cyclic nucleotide-gated channels to produce cytosolic free Ca^2+^; (ii) cGMP could be mediated through the activation of protein kinases (PKG); (iii) activation of PKG is a crucial step in NO-induced cyclic ADP-ribose (cADPR) synthesis. The cGMP/PKG/cADPR cascade has now been recognized as a mechanism where NO induces Ca^2+^ signals in various physiological processes. For the cGMP-independent mechanism, the effects of NO on Ca^2+^ homeostasis are through the S-nitrosylation of Ca^2+^ channels and transporters. Increasing evidence indicates that Ca^2+^ mediates plant adaptation to drought stress conditions. For example, Ca^2+^ has been shown to mediate stomatal movements in *Arabidopsis* under drought conditions [106]. Foresi et al. [107] reported that stomatal closure can be triggered by NO through regulation of Ca^2+^-sensitive potassium ion (K^+^) and chloride ion (Cl^−^) channels at the plasma membrane of guard cells. Other studies showed that Ca^2+^ and NO synergize to counteract drought stress effects by inducing initial root growth and development [25,68].

Similar to animal systems, the interactions between NO and melatonin in plants have been suggested to be both positive and antagonistic toward each other. Melatonin plays an important role in plant growth and modulation of defense responses [108]. Its protective roles in biotic and abiotic stresses have been widely investigated. Liang et al. [109] found that long-term exogenous application of melatonin improved nutrient uptake in apple plants under moderate drought conditions. The level of endogenous NO can be influenced by exogenous application of melatonin and vice versa. For example, exogenous application of melatonin increased NO production in alfalfa [110] and *Arabidopsis* [111]. It is believed that the interaction of NO and melatonin is through a cGMP-dependent pathway [112]. Although melatonin has been shown to positively regulate NOS-mediated NO biosynthesis in neuronal cells [113], as well as inducing mitochondrial NOS mRNA expression and protein synthesis [114], this molecule may suppress NOS activity during nitrosative stress [110]. Antagonistic interactions between melatonin and NO have been reported in root growth regulation in *Arabidopsis* during aluminum stress [115]. The authors found that exogenous melatonin could inhibit root growth in *Arabidopsis* during aluminum stress and decrease NO accumulation in roots.

PAs are polycationic molecules that are present in all living organisms. In plants, the most abundant PAs are putrescine (PUT), spermidine (SPD), and spermine (SPM). PAs are involved in various cellular processes, such as cell division and elongation, morphogenesis, and flowering [116]. PAs can also act as protective compounds to enhance plant tolerance against abiotic stresses [117]. Several studies demonstrated that PAs are involved in the production of H_2_O_2_ and NO [118,119]. This was evidenced by exogenously applying PAs on the drought-stressed plants like *Arabidopsis* [120], white clover [16], and cucumber seedlings [121]. The NO production was found to be increased in those plants, whereas this effect was reversed by the SPD synthase inhibitor (DCHA) [16]. In contrast, Montilla-Bascón et al. [122] reported that NO production was reduced in transgenic barley plants overexpressing the class 1 barley hemoglobin gene, *HvHb1*, an effective scavenger of endogenous NO. They found that the reduced NO level increased PA biosynthesis and ethylene biosynthetic genes, suggesting that NO might crosstalk with ethylene to regulate the expression of key enzymes in the PA biosynthetic pathways, such as arginine decarboxylase, ornithine decarboxylase, and methionine adenosyltransferase.

Taken together, these findings suggest a crosstalk between NO and other signaling molecules in drought stress adaptation. A better understanding of the interplay among these signaling molecules will undoubtedly enhance our current knowledge of the mechanisms triggered in plants against different stresses.

### 3.5. Crosstalk Between NO and Plant Hormones

Phytohormones play an important role in various processes, such as plant growth and development, as well as adaptation to biotic and abiotic stresses. Emerging evidence supports the notion that NO interplays with phytohormones, such as ABA, auxins, cytokinins (CKs), and ethylene, to regulate these biological processes [123]. For instance, NO has been found to crosstalk with ABA, jasmonic acid (JA), salicylic acid (SA), and CKs to mitigate the adverse effect of drought stress.

**Figure 4:**
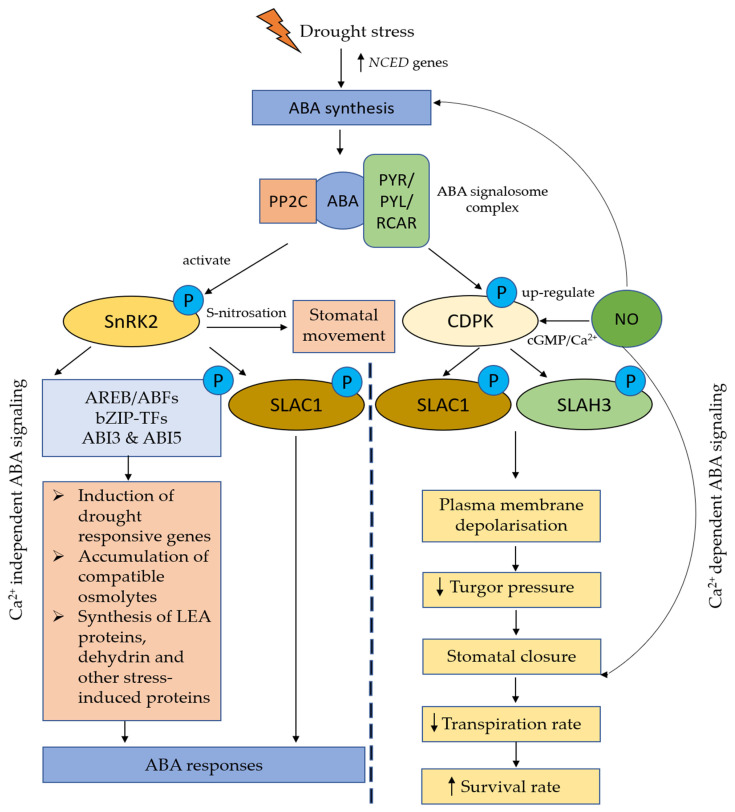
Schematic diagram showing crosstalk between abscisic acid (ABA) and nitric oxide (NO) under drought stress. Drought stress activates the regulation of *9-cis-epoxycarotenoid dioxygenase* (*NCED*) gene to enhance biosynthesis of ABA at leaf vascular parenchyma cells, which are the primary sites for ABA biosynthesis. ABA molecule binds to the pyrabactin resistance1/PYR1-like/regulatory components of ABA (PYR/PYL/RCAR) receptors and protein phosphatase 2C (PPC2) to form ABA signalosome complexes. These ABA signalosome complexes phosphorylate either (I) sucrose nonfermenting 1 (SNF1)-related protein kinase 2 (SnRK2) in Ca^2+^ independent ABA signaling pathway or (II) calcium-dependent protein kinase (CDPK) in Ca^2+^ dependent ABA signaling. In Ca^2+^ independent ABA signaling pathway, SnRK2 proteins phosphorylated AREB/ABF, b-ZIP, ABI3, and ABI5 transcription factors to induce expression of drought-responsive genes, accumulation of compatible osmolytes, as well as synthesis of LEA proteins, dehydrin, and other stress-induced proteins or ion channels (e.g., SLAC1), and subsequently trigger ABA responses. In Ca^2+^ dependent ABA signaling pathway, CDPKs are activated by NO. The increase of Ca^2+^ and phosphorylated ion channels [SLAC1 and S-type anion channel 3 (SLAH3)] trigger a cascade of ABA responses. These include plasma membrane depolarization, decrease of turgor pressure, stomatal closure, and reduction of transpiration rate.

ABA, an isoprenoid class of plant hormones, is well known as an antistress phytohormone which can rapidly accumulate under environmental stresses [124]. It is well-known that ABA can stimulate stomatal closure. Stomatal closure is one of the earliest plant responses to drought conditions. The purpose of the closure of stomata is to reduce the transpiration rate and ion leakage, which in turn decreases the rate of photosynthesis [76]. When plants are exposed to drought stress, a significant amount of ABA is produced. Once the ABA molecules reach guard cells via the ABC transporter, they bind to the pyrabactin resistance1/PYR1-like/regulatory components of ABA (PYR/PYL/RCAR) receptors to trigger a complex signaling network that leads to the stomatal closure (Figure 4). This signaling network involves the participation of several signaling molecules, such as Ca^2+^, NO, and H_2_O_2_; protein kinases; and transcription factors. Previous reports demonstrated that NO generation is essential for ABA-induced stomatal closure and that ABA can increase NO generation in the guard cells. There are numerous NO targets that have been identified in ABA-regulated signal transduction. These include plasma membrane calcium-dependent anion and K+ channels, protein phosphatase 2C (PPC2), and sucrose non fermenting 1 (SNF1)-related protein kinase 2.6 (SnRK2.6). It was shown that NO activated plasma membrane calcium-dependent anion channels but deactivated the inward-rectifying K+ channels in the guard cells [125], resulting in the loss of turgor pressure that causes stomatal closure [126]. Desikan et al. [127] reported that PP2C mutants (ABA-insensitive abi1-1 and abi2-1) generated NO in response to ABA but failed to close the stomata in response to NO, suggesting that PP2C might be downstream of NO in the ABA signaling cascade. The interaction between NO and ABI1 might be mediated by the GC/cGMP pathway [128]. Wang et al. [129] reported that NO negatively regulates ABA signaling in guard cells by inhibiting SnRK2.6 through S-nitrosylation. Several ABA-responsive genes have also been reported to be affected by NO treatment. These include *AtHVA22H* [130], *WRKY TF ABO3* [131], and *AtWRKY62* genes [132]. Taken together, both NO and ABA are important plant signaling molecules that work together to regulate redox balance and homeostasis against drought stress.

JA is a lipid-based hormone signal. It is an important endogenous regulator that controls various processes, including seed germination, senescence, and stress adaptation [133]. It is known that JA triggers a cascade of reactions, but only a few of them have connections with NO signaling. Evidence shows that JA could enhance NO synthesis in the guard cell, and the synergistic effect of JA and NO is able to induce stomatal movement and stomatal closure [134]. The JA- and NO-induced stomatal closure can be reversed by the NO scavenger. Exogenous application of NO induces the expression of JA biosynthesis-related genes, including 12-oxophytodienoate reductases (*OPR1*, *OPR2*, and *OPR3*) and lipoxygenases (*LOX3* and a putative lipoxygenase protein) [135]. Jian and Wu [136] reported that exogenously supplied methyl jasmonate could induce a rapid production of NO in *Taxus* cell cultures. They also found that other stress indicators, such as H_2_O_2_, MDA content, lipoxygenase, and PAL activities, were also increased after methyl jasmonate treatment. NO and JA show a synergistic relationship during drought conditions. For example, exogenous JA was found to enhance the drought tolerance in wheat by inducing NO production and upregulating the activity of the ascorbate-glutathione (AsA-GSH) cycle [137]. These findings show the interplay of NO and JA signaling in stress responses and adaptation; however, examination of the mechanism of these molecules in response to drought stress is scarce and requires further investigation.

Like ABA and JA, SA plays an important role in plant biological processes. Its relationship with NO, both synergistic or antagonistic, has been reported. SA was found to induce the production of NO in Arabidopsis [138] but reduce the NO production in tomato root tips [139,140]. This antagonistic effect of NO and SA might be influenced by the concentration of SA [138]. Recent studies indicated that NO and SA were able to enhance plant growth in terms of shoot length; biomass accumulation; leaf number and area; chlorophyll, carotenoid, and flavonoid content [51], in addition to inducing stomatal closure [141].

CKs play significant roles in plant growth and developmental processes, including cell division, leaf senescence, and chloroplast biogenesis [142]. A possible involvement of NO in cytokinin signal transduction was first reported in *Amaranthus caudatus* seedlings, where the production of the red pigment betalaine was influenced by not only CKs but also NO [143]. Since then, many reports have shown that NO production could be triggered by CKs [144,145]. Similar to NO, both CKs and auxins have been shown to regulate stomatal behavior. However, it is yet to be determined if cytokinin- and auxin-induced stomatal opening is related to NO in guard cells. It was reported that NO acts downstream of CKs [145]. The interactions of NO and CKs are, however, complex and may be synergistic and antagonistic. In maize, the combination of NO and CKs was able to enhance photosynthetic performance in response to drought stress [144]. In contrast, Xiao-Ping and Xi-Gui [146] reported that CKs reduced NO levels in the SNP-treated guard cells in light and dark conditions, thereby promoting reopening of closed stomata. Their effects might be varied in regard to plant species and experimental approach. For instance, NO positively regulates the expression of CK biosynthetic genes (*IPT1* and *ZR*) in the leaf tissues of *Gossypium hirsutum* [147], but NO-induced CKs reduce the NO level in *Vicia faba* [146]. Further research on this subject could help to further our understanding of how CKs and NO interact to respond to environmental stimuli.

**Figure 5:**
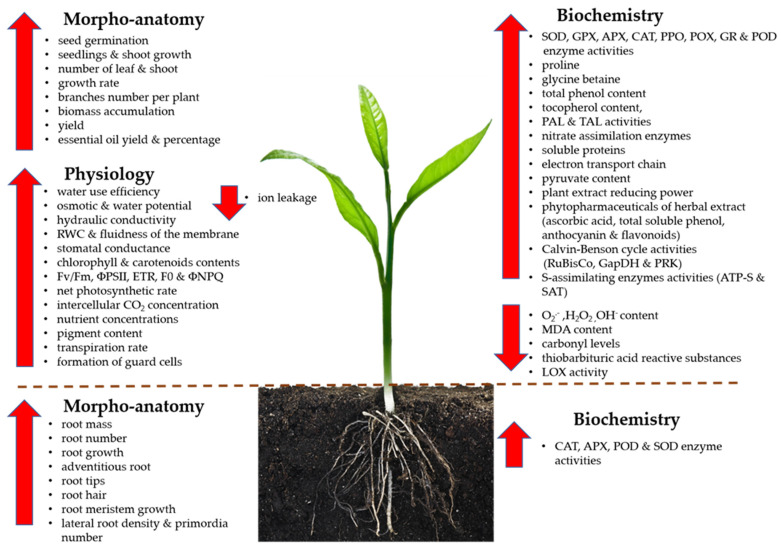
Overview of nitric oxide (NO) roles in alleviating drought stress at morpho-anatomical, physiological and biochemical levels in leaves and roots of a plant. ΦPSII, photosystem II; ΦNPQ, quantum yield of regulated energy dissipation; APX, ascorbate peroxidase; ATP-S, ATP-sulfurylase; CAT, catalase; ETR, electron transport rate; Fv/Fm, photochemical efficiency of photosystem Ⅱ; GAPDH, glyceraldehyde-3-phosphate dehydrogenase; GPX, glutathione peroxidase; GR, glutathione reductase; H_2_O_2_, hydrogen peroxide; LOX, lipoxygenase; POD and POX, peroxidases; PPO, polyphenol oxidase; O_2_^•−^, superoxide anion; O_2_, oxygen; OH^−^, hydroxyl radical; PAL, phenylalanine ammonia-lyase; PRK, phosphoribulokinase; RuBisCo, ribulose 1,5-bisphosphate carboxylase/oxygenase; SAT, serine acetyltransferase; SOD, superoxide dismutase; TAL, tyrosine ammonia-lyase.

At present, only a few studies have been carried out to investigate the relationship between phytohormones and NO in drought response. Our knowledge of their induction, production, and mechanism pathway of synergistic and antagonistic regulation remains limited, and continued efforts are needed to further elucidate how the pathways are interconnected. An overview of NO roles in alleviating drought stress at morphoanatomical, physiological, and biochemical levels in plants is illustrated in Figure 5.

## 4. NO-Responsive Proteins and Post-Translational Modifications

NO acts mainly by the PTMs of proteins, via *S*-nitrosylation, metal nitrosylation, carbonylation, and tyrosine nitration [148]. Generally, PTMs regulate the functions and stability of key molecules involved in important cellular processes, such as proteolytic degradation or subcellular localization. Defining NO-mediated PTM (NO-PTM) proteins and their mechanisms in the context of drought is thus essential for understanding how NO regulates biological functions.

*S*-nitrosylation is the most common NO-PTM that covalently links an NO group to a cysteine thiol of a protein to form SNOs [149]. Numerous *S*-nitrosylated proteins have been reported in plants. These *S*-nitrosylated proteins are able to regulate the activity of their target proteins, such as protein cleavage [150], signaling and ROS generation or scavenging [151], and disease resistance [152]. NO through S-nitrosylation of SnRK2 proteins was found to suppress the inhibitory effect of ABA on germination and cotyledon development [153]. In addition, the increased S-nitrosothiol content was associated with an improvement in initial seedling growth in common bean [25] and *Arabidopsis* [153]. Nitrosylation/denitrosylation of ascorbate peroxidase 1 (APX1) and histidine phosphotransfer protein 1 (AHP1) could regulate root development and architecture [154] and CK signaling [155], respectively. GSNO, formed by *S*-nitrosylation of the antioxidant glutathione, is a major regulator of NO-SNO signaling by acting as an NO reservoir in cells [156]. Emerging evidence indicates that SNOs may be the key players in NO-signaling pathways involved in plant immunity and response to abiotic stresses by modulating the antioxidant capacity in order to regulate the redox homeostasis [157,158,159]. Efforts should thus be made to identify the SNO-mediated processes and *S*-nitrosylated proteins under physiological or stress conditions.

Tyrosine nitration is the reaction of a nitrating agent with a tyrosine residue of any target protein that leads to the addition of a nitro group (NO_2_) to one of the two equivalent ortho carbons of the aromatic ring of tyrosine residues, resulting in 3-nitrotyrosine [156]. This process is mediated by peroxynitrite and nitrogen dioxide [160]. This modification was first thought to be irreversible, but emerging evidence suggests that denitrification may occur, either enzymatically or nonenzymatically [161]. Tyrosine nitration is an important redox-mediated PTM in plants, but only a few studies have been reported [162,163]. Efforts to use physiological approaches to investigate the biological role of this PTM in plant physiology are indispensable to better understand the impact of NO.

Metal nitrosylation involves the addition of a NO group to a transition metal, forming a metal-nitrosyl complex. In animals, guanylate cyclase and cytochrome *c* oxidase are examples of a protein whose function is regulated by metal nitrosylation [164]. In plants, reaction of hemoglobins with NO through metal nitrosylation has been reported to be part of a protection against NO burst [165].

Proteomic approaches have been widely used to identify proteins undergoing nitrosylation. We can now concomitantly identify the nitrosylated cysteines at the exact site of modification. The recent advances in proteomic technologies and mass spectrometry have substantially increased the number of identified proteins. More effort, however, must be put on enriching low abundance nitrosylated proteins, which can be due to either a low level of nitrosylation or simply to low abundance of the protein. Importantly, to better understand the functions of this PTM in plants, many identified *S*-nitrosylated proteins should be validated using candidate-specific approaches rather than proteomics analysis. Since the analysis of *S*-nitrosoproteome under drought conditions remains largely untapped, plant scientists should now investigate the underlying mechanisms and functional roles of protein nitrosylation and identify candidate proteins associated with water deficit. The molecular mechanisms controlling the nitrosylation level and the mechanisms of signal integration at the interface between the nitrosylation network and other PTMs and signaling molecules are among the topics that require further exploration [166].

## 5. Detection of NO: Challenges

The quantification of NO is technically challenging due to its unique chemical properties. NO is produced in subcellular compartments in a broad range of concentrations, from pico- to micro-molar. It diffuses freely across membranes and has a short half-life (seconds) in biological systems [167]. In addition, NO signaling displays a complicated temporal and spatial arrangements which further impedes its detection [167]. Due to its ubiquity and involvement in various intrasignaling processes, the analytical methods used for its detection and analysis should suit the biological model and take into account the distinct features of NO production and functions. For example, NO generation in barley infected by *Blumeria graminis* f. sp. *hordei* (powdery mildew) was reported by a subtle change in the induction of the epidermal or the formation of cell wall papillae, which can be very difficult to detect using only a gas-based NO detection system [168]. Due to these difficulties, questions have been raised regarding the roles of NO in certain biological processes and the specificity of the detection methods used.

Fluorescent probes have been widely used to detect cellular NO due to their excellent sensitivity and selectivity. Most fluorescent probes, however, not only bind to NO but also bind to its oxidation by-products, other reactive secondary species or divalent metals, such as dinitrogen trioxide (N_2_O_3_), NO_3_^−^, peroxidases, H_2_O_2_, ascorbic acid, dehydroascorbic acid, and calcium chloride [169]. One such example is diaminofluorescein (DAF) dyes (DAF-2 and DAF-2DA). DAF dyes have been commonly used to detect NO in plants because these dyes are relatively cheap and can be visualized using fluorescence microscopy [170]. The basic principle of the assay involves the hydrolysis of the diacetate groups on the DAF-2DA by cytosolic esterases to form the nonfluorescent compound, DAF-2. In the presence of NO, the DAF-2 compound is then converted to a fluorescent triazole derivative, DAF-2T, allowing researchers to localize NO production in the cells [171]. The main drawback of the DAF dye method is its tendency to react with other ROS, such as peroxidases and H_2_O_2_ [169]. Recently, an electrochemically based method using a homemade platinum- or iridium-based electrochemical microsensor has been reported to provide real-time detection of NO production in plant cell suspension cultures [167]. In addition, another electrochemical sensing microbundle that can simultaneously measure NO, H_2_O_2_, and pH under drought stress at various developmental stages was invented [172].

Chemiluminescence is a chemical assay used to detect NO based on NO reaction with ozone. Upon reacting with ozone, NO forms nitrogen dioxide at an excited state. As it decays into the ground state, it emits a photon that can be detected by a photomultiplier tube [173]. Since the photon emitted is proportional to the reacting NO, this allows a reliable estimation of NO. This method holds advantages over other NO detection methods due to its sensitivity (detection in the pmole range) and ability to directly measure NO in both aqueous and gaseous states [174]. The disadvantages of the chemiluminescence method include reading errors that could be introduced due to the sensitivity of the photomultiplier or changes in the light generated by the secondary source of light. Another disadvantage is that chemiluminescence only measures gaseous NO and no other forms of nitrogen or reactive nitrogen intermediates [170]. Thus, the considerable levels of NO that are oxidized in living tissues cannot be easily detected using this method.

“Spin-trapping” is a commonly used method involving the detection of a transient, reactive free radical using a high concentration of diamagnetic spin trap. This approach is known as electron paramagnetic resonance (EPR) spectroscopy (also known as electron spin resonance, ESR, or electron magnetic resonance, EMR) [175]. Since NO and superoxide are diatomic free radicals, their presence could be detected using this method. NO can be effectively measured in the gas phase using EPR; however, its signal is less sensitive in the liquid phase [176]. Since the steady-state level of NO is below the detectable limit of EPR, NO needs to be trapped in a more stable form to allow its proper detection. This is achieved by adding a reactive free radical to the double bond of a diamagnetic compound known as “spin trap,” forming a more persistent and stable secondary free radical (radical adduct) [177]. An example is nitrone 5,5-dimethyl-1-pyrroline N-oxide (DMPO) which is used to trap free radicals, such as superoxide and hydroxyl, to provide the radical adducts with a distinctive spectrum. Unlike superoxides, which are highly stable and allow direct measurement, NO depends on reaction with other free radicals or its ability to coordinate with iron ions for a more reliable measurement [178]. Although the use of EPR for NO detection is not as direct or sensitive as other detection methods (such as chemiluminescence or electrochemical methods), its application is more effective under certain conditions. For example, EPR is not sensitive to magnetic susceptibility effects (unlike nuclear magnetic resonance (NRM) spectroscopy) and does not need optical transparency, enabling the measurement of cells or tissues in turbid solutions or solid form.

Developing efficient and sensitive methods of detection is clearly indispensable for the accurate detection and quantification of NO. Mur et al. [168] suggested the use of more than one technique to detect NO. In a study by Bright et al. [179], a combination of the DAF dye technique and EPR spectroscopy was applied to measure NO production when hydrating pollen. Besson-Bard et al. [180] combined the NO probe DAF-2 and an electrochemical method to detect the role of cryptogein, a fungal elicitor, in increasing NO production under biotic stress in tobacco. A similar approach was used to measure NO production in tobacco suspension cells [170]. The recent advances in quantum cascade laser-based spectroscopy have offered new opportunities to measure gaseous NO based on the absorption of laser light by the NO molecules and provide online planta measurements of the dynamics of NO production. Despite its sensitivity, this detection technique also has its flaws, including unintended interferences with another species, unreliable sensitivity, and indirect measurement taken from the secondary NO species [122].

## 6. Conclusions and Perspectives

NO is an important signaling molecule in drought response and adaptation. Although a substantial amount of data exists to support the role of NO in response to drought, many questions remain unanswered regarding NO regulation and its biological roles. How is NO synthesized? Where is NO accumulated? How does exogenous NO interact with endogenous NO? Moreover, it is important to investigate how NO regulates crosstalk between other signaling molecules (H_2_S, ROS, and ABA), phytohormones (JA, SA, and ethylene), organic compounds (PAs), calcium, and pleiotropic molecule (melatonin) in alleviating drought stress effects. Given that NO signaling is a complex network, the use of bioinformatics tools to process and analyze the omics dataset is crucial. In terms of experiments, the design and methodology should be scientifically sound and conducted in a careful manner to obtain reliable results. The use of whole mature plants instead of seedlings, protoplasts, and detached leaves or whole plants in growth chambers should be encouraged to decipher the role of NO in response to drought under field conditions in order to achieve practical prospects.

Unfortunately, there are no entirely satisfactory detection methods for NO. Each current method has its limitations. Developing efficient and sensitive methods for NO detection is thus indispensable for quantifying and tracing NO in vivo in different tissues and at different times. With the advancement of technology, it is expected that a significant amount of data from plant NO research may be revisited in the near future, helping to better interpret NO function.

## Data Availability

Not applicable.
